# The feasibility of differentiating colorectal cancer from normal and inflammatory thickening colon wall using CT texture analysis

**DOI:** 10.1038/s41598-020-62973-1

**Published:** 2020-04-14

**Authors:** Xiao Wang, Mingyuan Yuan, Honglan Mi, Shiteng Suo, Khalid Eteer, Suqin Li, Qing Lu, Jianrong Xu, Jiani Hu

**Affiliations:** 1 Department of Radiology, Renji Hospital, School of Medicine, Shanghai Jiao Tong University, 160 Pujian Rd, Shanghai, 200127 China; 2 Department of Radiology, Shanghai Pudong Hospital, Fudan University Pudong Medical Center, 2800 Gongwei Road, Pudong, Shanghai 201399 China; 3 Department of Radiology, Affiliated Zhoupu hospital, Shanghai university of medicine & health Sciences College, 1500 Zhouyuan Road, Pudong New District, Shanghai, 201318 China; 4 Department of Radiology, Wayne State University School of Medicine, Detroit, MI 48201 USA

**Keywords:** Colon cancer, Inflammatory bowel disease

## Abstract

To investigate the diagnostic value of texture analysis (TA) for differentiating between colorectal cancer (CRC), colonic lesions caused by inflammatory bowel disease (IBD), and normal thickened colon wall (NTC) on computed tomography (CT) and assess which scanning phase has the highest differential diagnostic value. In all, 107 patients with CRC, 113 IBD patients with colonic lesions, and 96 participants with NTC were retrospectively enrolled. All subjects underwent multiphase CT examination, including pre-contrast phase (PCP), arterial phase (AP), and portal venous phase (PVP) scans. Based on these images, classification by TA and visual classification by radiologists were performed to discriminate among the three tissue types. The performance of TA and visual classification was compared. Precise TA classification results (error, 2.03–12.48%) were acquired by nonlinear discriminant analysis for CRC, IBD and NTC, regardless of phase or feature selection. PVP images showed a better ability to discriminate the three tissues by comprising the three scanning phases. TA showed significantly better performance in discriminating CRC, IBD and NTC than visual classification for residents, but there was no significant difference in classification between TA and experienced radiologists. TA could provide useful quantitative information for the differentiation of CRC, IBD and NTC on CT, particularly in PVP images.

## Introduction

Colorectal cancer (CRC) is one of the most commonly diagnosed and deadly cancers worldwide^1^. The risk of CRC is increased in patients with inflammatory bowel disease (IBD), including ulcerative colitis (UC) and Crohn’s disease (CD), compared with that of sporadic CRC, especially in IBD patients with long-term colitis, strictures, fistulae, and right-sided colonic disease^2^. Although the incidence of CRC in IBD patients accounts for only 1–2% of all CRC cases, a recent population-based study showed that CRC accounted for 10–15% of all IBD-related deaths^3^. Therefore, CRC screening and early detection in IBD patients may reduce the morbidity and mortality rates of CRC in patients with IBD^4^.

Regular monitoring by endoscopy may allow the early detection of CRC. However, endoscopy usually involves sedation and has associated risks, including perforation and bleeding, especially in patients with active IBD^5^. Compared with colonoscopy, computed tomography (CT) is a promising method for CRC screening due to the lower rate of test-related complications, the ability to assess the patient for perforating complications of IBD, and the ability to determine the extent and severity of CRC and IBD^6^.

On CT images, patterns of wall thickening are helpful for differential diagnosis, with heterogeneous and asymmetrical focal thickening indicating malignancies and homogeneous and symmetrical regular thickening suggesting benign or well-differentiated tumours^2^. For experienced gastrointestinal radiologists, it is not very difficult to distinguish CRC from IBD, but for less experienced radiologists or residents, there are still some challenges. Moreover, colonic thickening in IBD, including UC and CD, can often present as asymmetrical and therefore mimic CRC, which can result in difficulty in distinguishing between the two processes^7^. Furthermore, acute and chronic changes of the colorectal mucosa make it difficult to distinguish tumours from the underlying IBD by CT^8^. Therefore, improving the sensitivity and discriminative power of CT images for the detection and differentiation of CRC is very important, particularly in patients with colonic mural thickening with a possible underlying malignancy and an overlap in thickness^9^.

As one part of the growing field of radiomic analysis, CT texture analysis (CTTA) is a new imaging post-processing technique^10^ in which texture features are computed from the image intensity distribution of pixels/voxels in two-/three-dimensional (2D/3D) space and used to characterize the texture types and thus the underlying structures of the objects embedded in the CT image^11,12^. An increasing number of studies have shown that CTTA is a potentially useful tool in tumour imaging^13–15^. In one study^16^, the feasibility of using CT texture parameters to differentiate of colorectal signet-ring cell carcinoma and adenocarcinoma have been shown. To the best of our knowledge, employing texture feature analysis techniques for differentiating CRC from IBD has not been widely reported in this field. Consequently, the aim of this study was to examine not only the feasibility of using texture analysis (TA) to differentiate CRC from non-tumour tissues but also the potential diagnostic power of CTTA in the differential diagnosis of tumorous and non-tumorous colonic disease compared with human visual classification.

## Results

**Figure 1:**
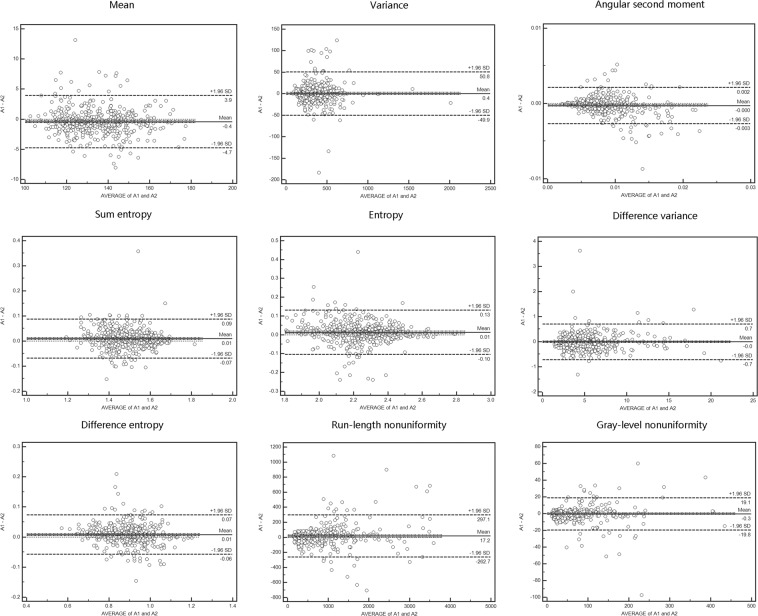
The reproducibility of the extracted texture features between readings A1 and A2.

**Figure 2:**
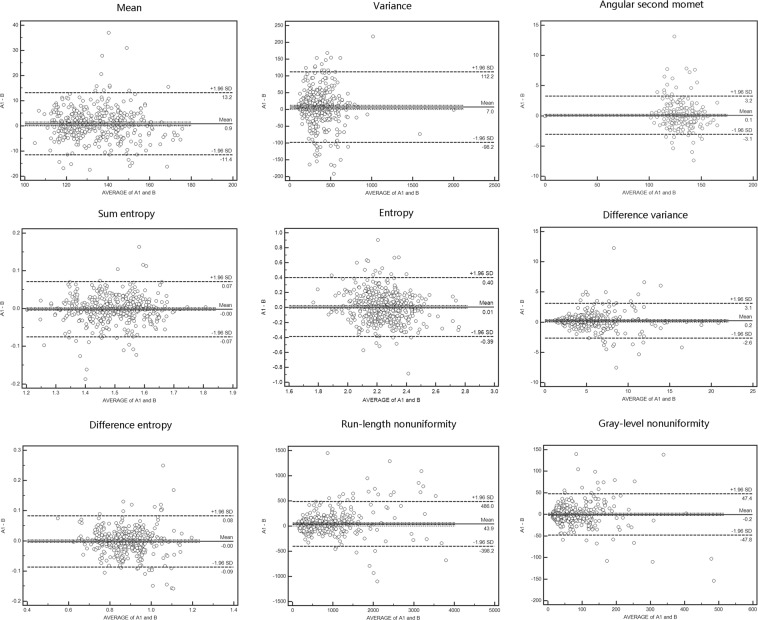
The reproducibility of the extracted texture features between readings A1 and B.

The mean pixels in the measured ROIs and the mean thickness of the colon wall or lesion are summarized in Table 1, there were no significant differences among the three readings obtained by the two readers in the pre-contrast phase (PCP), arterial phase (AP), or portal venous phase (PVP) images (all p > 0.05). The measurements of the pixels in the region of interest (ROI) and the colon wall thickness also showed substantial agreement between readings and between readers (Table 2, all intra-class correlation coefficients (ICC) > 0.75). The reproducibility of the nine extracted texture features showed better agreement between readings A1 and A2 than between readings A1 and B. (Figs. 1 and 2, see Supplementary Table A).

**Table 1:** The number of pixels in ROI and the thickness of colon wall.

	Scan phase	Item	A1	A2	B	*F*	*P*
The pixels in ROI	PCP	CRC	647.96 ± 42.41	637.14 ± 34.42	675.45 ± 39.60	0.16	0.848
		IBD	629.46 ± 37.66	632.97 ± 30.77	607.51 ± 29.94	0.07	0.930
		NTC	513.13 ± 19.34	540.10 ± 23.61	557.73 ± 19.71	0.80	0.449
	AP	CRC	724.59 ± 41.65	713.89 ± 37.01	730.48 ± 35.79	0.03	0.969
		IBD	739.65 ± 36.08	728.45 ± 25.56	723.53 ± 27.27	0.03	0.975
		NTC	623.60 ± 20.57	629.52 ± 25.81	626.16 ± 27.10	0.01	0.990
	PVP	CRC	757.03 ± 41.94	755.17 ± 39.25	753.06 ± 30.38	0.01	0.998
		IBD	694.12 ± 31.46	688.32 ± 21.67	680.29 ± 30.58	0.03	0.975
		NTC	628.98 ± 25.72	636.05 ± 25.84	647.22 ± 27.83	0.09	0.917
The mean thickness of colonic wall(mm)	PCP	CRC	9.03 ± 2.51	8.78 ± 1.82	8.72 ± 2.48	0.33	0.172
		IBD	8.49 ± 1.68	8.62 ± 1.48	8.66 ± 1.74	0.12	0.891
		NTC	8.22 ± 1.27	8.24 ± 1.55	8.21 ± 1.28	0.01	0.991
	AP	CRC	8.78 ± 2.78	8.26 ± 1.95	8.75 ± 2.69	0.87	0.419
		IBD	8.06 ± 1.91	8.10 ± 1.59	8.17 ± 1.86	0.04	0.960
		NTC	8.05 ± 1.28	7.67 ± 1.48	7.88 ± 1.32	1.39	0.253
	PVP	CRC	8.66 ± 2.86	8.53 ± 1.92	8.63 ± 2.63	0.05	0.953
		IBD	8.23 ± 2.00	8.40 ± 1.59	8.49 ± 1.78	0.12	0.890
		NTC	7.77 ± 1.32	7.87 ± 1.39	7.83 ± 1.25	0.09	0.917

Note: Significant *p* value < 0.05; ROI, region of interest; CRC, colorectal cancer; IBD, inflammatory bowel disease; NTC, normal thickening colon; PCP, precontrast phase; AP, arterial phase; PVP, portal vein phase; A1, reader A first read; A2, reader A second read; B, reader B.

**Table 2:** ICC value of intra- and inter observer agreement for the measurement of the pixels in ROI and the thickness of colon wall.

	Scan phase	Item	A1 vs A2	A1 vs B
The pixels in ROI	PCP	CRC	0.78	0.77
		IBD	0.77	0.76
		NTC	0.78	0.75
	AP	CRC	0.79	0.78
		IBD	0.79	0.78
		NTC	0.76	0.77
	PVP	CRC	0.80	0.76
		IBD	0.78	0.76
		NTC	0.80	0.79
The mean thickness of bowel wall	PCP	CRC	0.81	0.80
		IBD	0.82	0.78
		NTC	0.79	0.77
	AP	CRC	0.80	0.78
		IBD	0.77	0.78
		NTC	0.83	0.80
	PVP	CRC	0.86	0.78
		IBD	0.89	0.79
		NTC	0.90	0.81

Note: ICC, intraclass correlation coefficients; ROI, region of interest; CRC, colorectal cancer; IBD, inflammatory bowel disease; NTC, normal thickening colon wall; PCP, precontrast phase; AP, arterial phase; PVP, portal vein phase; A1, reader A first read; A2, reader A second read; B, reader B.

The frequencies of texture features that were selected based on the Fisher coefficients, minimization of both classification error probability (POE), average correlation coefficients (ACC), and mutual information coefficients (MI) are listed in Table 3. For the discrimination of IBD from normal thickened colon wall (NTC) and IBD from CRC, the selected features were predominantly derived from the run-length matrix (RLM), the co-occurrence matrix (COM) and Histogram, while for the discrimination of CRC from NTC and CRC from the other two tissues (IBD and NTC), the features were mostly extracted from COM, RLM and wavelet.

**Table 3:** The frequencies of feature category to be selected.

Texture parameter groups	PCP	AP	PVP
CRC vs. IBD			
Histogram (n = 9)	2	4	6
COM (n = 220)	8	17	9
RLM (n = 20)	15	6	14
GrM (n = 5)	0	0	0
ARM (n = 5)	2	1	0
Wavelet (n = 16)	3	2	1
IBD vs. NTC			
Histogram (n = 9)	2	4	3
COM (n = 220)	18	21	24
RLM (n = 20)	8	2	2
GrM (n = 5)	0	0	0
ARM (n = 5)	1	1	0
Wavelet (n = 16)	1	2	1
CRC vs. NTC			
Histogram (n = 9)	0	1	1
COM (n = 220)	6	14	14
RLM (n = 20)	12	6	15
GrM (n = 5)	5	3	0
ARM (n = 5)	0	0	0
Wavelet (n = 16)	7	6	0
CRC. vs. IBD vs. NTC			
Histogram (n = 9)	0	2	1
COM (n = 220)	3	13	13
RLM (n = 20)	14	5	15
GrM (n = 5)	6	3	0
ARM (n = 5)	0	0	0
Wavelet (n = 16)	7	7	1

Note: COM, the co-occurrence matrix; RLM, the run-length matrix; GrM, the absolute gradient; ARM, the autoregressive model; CRC, colorectal cancer; IBD, inflammatory bowel disease; NTC, normal thickening colon wall; PCP, precontrast phase; AP, arterial phase; PVP, portal vein phase.

**Figure 3:**
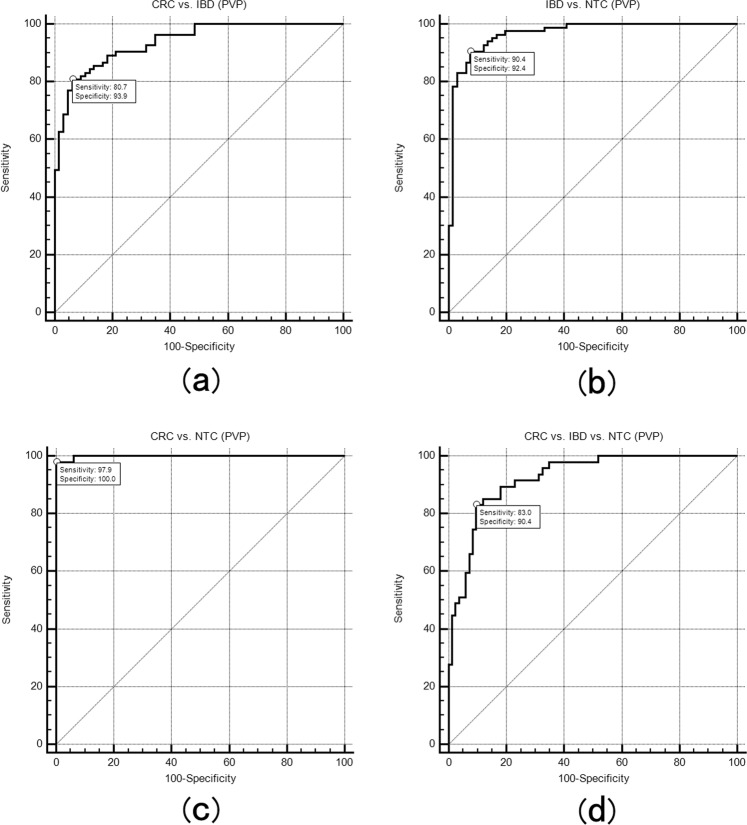
Receiver operating characteristic (ROC) curves showing the texture features classification performances in portal vein phase (PVP). CRC vs. IBD (**a**), IBD vs. NTC (**b**), CRC vs. NTC (**c**) and CRC vs. IBD vs. NTC (**d**). The area under ROC curves analysis provided in Supplementary Table B.

Tissue texture-based classification results for all CT scan phases are shown in Table 4. The performance of the nonlinear discriminant analysis (NDA) classifier showed excellent classification results (misclassification rate (MCR) 1.66–31.84%) for all classification groups regardless of CT scan phase or feature extraction method compared to the other two classifiers (Principal component analysis (PCA); Linear discriminant analysis (LDA)). Tissue classification for discriminating three tissues (CRC vs. NTC vs. IBD) achieved relatively poorer results (MCR, 12.61–31.84%) than that for discriminating any two tissues (CRC vs. NTC, IBD vs. NTC, or CRC vs. IBD; MCR, 1.66–14.04%). By using NDA classifiers with subset feature extraction methods (Fisher coefficient, POE + ACC and MI coefficient), the MCR for the classification of two or three tissues decreased from PCP images to AP images to PVP images. The receiver operating characteristic (ROC) curve analysis results for PVP are presented in Fig. 3. In the comparison CRC vs. IBD; IBD vs. NTC; CRC vs. NTC; CRC vs. IBD vs. NTC, the sensitivity, specificity and area under curve (AUC) value were 80.72%, 93.86%, 0.94 ± 0.02 (95% confidence interval: 0.89, 0.97); 90.38%, 92.41%, 0.97 ± 0.01 (95% confidence interval: 0.92, 0.98); 97.92%, 100%, 0.98 ± 0.01 (95% confidence interval: 0.96, 0.99); 83.04%, 90.43%, 0.92 ± 0.02 (95% confidence interval: 0.86, 0.96), respectively. The AUC values of each extracted texture feature were listed in Supplementary Table B.

**Table 4:** The misclassification rate (%) for discrimination between CRC, IBD and NTC on three CT scan phase images with three different classifiers based on texture features selected by Fisher, POE + ACC and MI methods.

Scanning phase	Feature selection method	PCA	LDA	NDA
**CRC vs. IBD**				
PCP	Fisher	20.43	19.35	9.68
	POE + ACC	20.43	19.35	6.45
	MI	18.98	17.20	6.45
AP	Fisher	16.35	23.66	8.60
	POE + ACC	16.35	19.13	8.60
	MI	15.61	19.13	4.30
PVP	Fisher	13.05	15.66	5.38
	POE + ACC	13.05	15.66	5.38
	MI	14.20	14.20	6.45
**IBD vs. NTC**				
PCP	Fisher	23.98	18.71	14.04
	POE + ACC	23.98	18.71	14.04
	MI	17.54	15.20	9.36
AP	Fisher	21.05	19.30	2.76
	POE + ACC	21.05	19.30	7.02
	MI	24.56	25.73	11.70
PVP	Fisher	22.35	24.71	8.82
	POE + ACC	22.35	14.71	5.29
	MI	15.29	15.88	6.47
**CRC vs. NTC**				
PCP	Fisher	10.44	10.99	6.59
	POE + ACC	23.08	27.47	13.74
	MI	7.69	12.64	4.95
AP	Fisher	15.38	15.38	6.59
	POE + ACC	20.33	12.09	4.95
	MI	20.33	18.13	7.14
PVP	Fisher	10.50	6.08	1.66
	POE + ACC	10.50	6.08	1.66
	MI	6.63	6.63	2.76
**CRC vs. IBD vs. NTC**				
PCP	Fisher	23.77	26.46	21.52
	POE + ACC	32.29	47.98	31.26
	MI	26.91	30.94	19.28
AP	Fisher	29.15	25.11	19.28
	POE + ACC	30.04	29.60	19.28
	MI	28.70	34.08	31.84
PVP	Fisher	19.37	22.97	12.61
	POE + ACC	19.37	22.97	12.61
	MI	36.94	39.19	30.63

Note: CRC, colorectal cancer; IBD, inflammatory bowel disease; NTC, normal thickening colon wall; POE + ACC, Probability of classification error and average correlation; MI, Mutual information coefficients; PCA, Principal component analysis; LDA, Linear discriminant analysis; NDA, Nonlinear discriminant analysis; PCP, precontrast phase; AP, arterial phase; PVP, portal vein phase.

The average MCR for the visual classification results and the average MCR for the CTTA classification results by the NDA classifier are summarized in Table 5. As shown in this table, the MCR for CTTA between readers C and D showed no significant difference except between CRC and NTC in PVP images. However, the MCR obtained by CTTA with the NDA classifier was significantly lower than that obtained by visual classification by readers E and F (p < 0.05), except for the MCR obtained from three-tissue classification in AP and PVP images. The MCR obtained from the three-phase CT scan decreased from the PCP images to the AP images and the PVP images, regardless of whether visual classification or textural classification methods were used.

**Table 5:** The comparison of the misclassification rate (MCR) between visualization and texture analysis with classifier NDA for discrimination between CRC, IBD and NTC on three phase CT scan images.

Scanning phase	MCR (%) of TA	MCR (%) of Reader C, D	MCR (%) of Reader E, F	TA vs. Reader C, D		TA vs. Reader E, F	
				*t*	*p*	*t*	*p*
**CRC vs. IBD**							
PCP	7.53 ± 1.86	15.85 ± 4.56	31.68 ± 1.46	3.613	0.069	15.191	<0.001
AP	7.17 ± 2.48	12.23 ± 1.39	27.60 ± 1.29	2.440	0.135	10.368	0.002
PVP	5.74 ± 0.36	9.53 ± 2.72	16.59 ± 1.19	2.769	0.109	19.112	<0.001
**IBD vs. NTC**							
PCP	12.48 ± 2.70	18.74 ± 1.45	26.81 ± 1.99	2.646	0.118	6.315	0.008
AP	7.16 ± 4.47	14.52 ± 1.89	22.76 ± 1.53	2.578	1.124	4.550	0.019
PVP	6.86 ± 1.80	11.81 ± 1.44	17.43 ± 1.57	2.971	0.097	7.714	0.007
**CRC vs. NTC**							
PCP	8.43 ± 4.67	14.48 ± 1.18	26.47 ± 1.19	2.929	0.109	5.098	0.015
AP	6.23 ± 1.14	10.53 ± 2.65	25.08 ± 2.05	3.866	0.089	13.717	<0.001
PVP	2.03 ± 0.64	9.15 ± 1.72	15.21 ± 1.90	3.951	0.007	11.896	0.001
**CRC vs. IBD vs. NTC**							
PCP	24.02 ± 6.37	32.04 ± 1.59	40.77 ± 1.12	2.102	0.175	3.501	0.039
AP	23.47 ± 7.25	27.86 ± 2.32	35.82 ± 1.20	1.038	0.408	2.270	0.107
PVP	18.62 ± 10.40	21.19 ± 1.65	26.51 ± 1.65	0.446	0.699	1.011	0.386

Note: CRC, colorectal cancer; IBD, inflammatory bowel disease; NTC, normal thickening colon wall; NDA, Nonlinear discriminant analysis; PCP, precontrast phase; AP, arterial phase; PVP, portal vein phase; TA, Texture analysis. *p* < 0.05 indicates significant.

## Discussion

The hallmark of colonic tumorous and non-tumorous processes on CT is mural thickening. However, this is a non-specific feature that reduces the diagnostic value of CRC. The goal of the present study was to test the feasibility of the classification of CRC, IBD and NTC on routine CT images based on TA. To the best of our knowledge, there have been no similar reports on this topic before. Our results suggest that TA may be used to distinguish CRC from IBD or NTC. Our experimental data show that in PVP images, the accuracy of classification was as high as 94.3% for CRC vs. IBD, 98.0% for CRC vs. NTC, 93.1% for IBD vs. NTC, and 81.4% for CRC vs. IBD vs. NTC. Our results indicate that it is sufficient to calculate the texture features according to the standard spatial resolution of routine CT images, such that tumours can be distinguished from non-tumours in most cases (up to 81.4% for NDA/artificial neural network (ANN)).

On comparison of the three-phase CT scans, we found that PVP images allowed better differentiation of CRC versus IBD or NTC than AP images or PCP images. These results may be due to different histological components and enhancement patterns of the colon wall in CRC, IBD, and NTC. Although abnormally thickened colon walls showed similar attenuation in PCP, the classification performance obtained by TA with the NDA/ANN classifier showed fairly high accuracy (MCR, 7.53–12.48%) in all three comparisons (CRC vs. IBD, CRC vs. NTC, and IBD vs. NTC), which indicated that TA could detect differences in tissue type among CRC, IBD and NTC because of the complex histological components. Thus, after administration of contrast agent, these different complex histological components of the tissues would lead to higher classification accuracy with PVP images (MCR, 2.03–6.86%) for discriminating CRC vs. IBD, CRC vs. NTC, and IBD vs. NTC.

In our study, the texture features based on the RLM and COM were more often selected than other categories of features, regardless of what feature selection method or CT sequence was used. RLM-based features show the same grey-scale value for a single image in a given direction. The use of textural differences to distinguish between these diseases has been demonstrated, primarily owing to differences in the attenuation of pathological and healthy tissues^17^. In agreement with published research, our study demonstrates that RLM-based features could distinguish normal from abnormally thickened colon wall tissue on CT images. Texture features based on the COM show attenuation (CT value) changes as distance increases and reflect whether the attenuation of the ROI is uniform. In this study, we found that a high frequency of COM-based features should be chosen, which is consistent with other studies based on MR images^11,18^. This may be because the density of CRC or IBD is relatively uniform and that of NTC is heterogeneous on CT images, especially on PVP contrast-enhanced CT images^19,20^. This may also be caused by the large number (n = 220) of features based on the COM, and some of them may exhibit perfect potential for differentiation. It must be noted that there were some combinations of texture features that could not identify CRC, IBD, and NTC simultaneously. This result suggests that TA may be most useful currently to narrow the differential diagnosis to two diseases.

CT and MRI have a similar diagnostic accuracy for IBD^21–23^, but previous studies have shown that MRI has higher accuracy than CT in the diagnosis of CRC, with an overall accuracy of 83.9% for CT and 90.5% for MRI^24^. This was mainly because CT had a lower resolution in soft tissues and could not effectively distinguish the layers of the bowel wall^25^. In this study, using CT images in combination with texture feature analysis, the average diagnostic accuracy in differentiating CRC from IBD was 94.3% when the MCR used the NDA converting algorithm. These findings suggest that CT imaging combined with texture feature analysis is comparable to or possibly even better than MRI. Moreover, CT was less time consuming and produced fewer air artefacts. Because CT examinations have some advantages, such as wide availability, high speed, and low cost, CT is preferred for CRC screening, particularly in patients with IBD. Furthermore, it is possible to improve the diagnostic performance of CT if images from multiple scanning phases are combined with TA.

The comparison of visual-based classification and texture feature-based classification clearly shows no significant difference in most results between TA and readers C and D. It is important to note that readers C and D in this study had more than 10 years of experience. Moreover, TA had just ‘read’ one CT image in one scanning phase, but the readers had read all CT images in one scanning phase, which provided more diagnostic information, such as the degree of bowel wall thickening, contrast-enhancement characteristics, and characteristics of lymphatic and adipose tissues surrounding the bowel wall. In contrast, most results between TA and readers E and F showed significant differences, which indicated that for less experienced radiologists or residents, TA could be helpful for improving the diagnostic skill level and increasing the diagnostic accuracy. Furthermore, TA will improve the work efficiency and reduce the work burden of experienced radiologists. Many artificial intelligence (AI) techniques have been implemented based on TA^26–28^, and our research provides a basis for the future application of AI techniques in intestinal diseases.

Our study has several limitations. First, images from different enhanced CT phases were analysed separately. If a comprehensive evaluation of multiple phases of images is performed, the identification accuracy might be enhanced. Second, the size of inflammatory lesions in IBD is relatively small, and the selection of NTC may involve bias; however, our data show excellent intra- and fairly good inter-class agreement for TA in CRC, IBD and NTC (Figs. 1 and 2, see Supplementary Table A). Our future research will include more cases to reduce bias. Third, previous work has shown that three-dimensional TA is superior to a two-dimensional approach in the discrimination of pathological tissues^29^. In our study, we used two-dimensional TA on ROIs in three axial sections rather than three-dimensional analysis on the entire thickened colon volume, which would be less sensitive to lesions or colon wall variations. We chose to use two-dimensional CT images, which are easy and convenient to access in the clinic, and some previous work has not verified whether three-dimensional TA is better than two-dimensional TA^30^. Our future research will focus on increasing the number of cases and comparing the test efficiency between three-dimensional TA and two-dimensional TA. Furthermore, we did not divide the enrolled population into training and testing groups because we mainly focused on the general feasibility of TA classification based on routine CT images. In addition, a separate training group is not needed in the K-nearest neighbor (k-NN) and the ANN classifier in the MaZda program by leave-one-out testing method^31^.

In conclusion, we found that TA is useful for differentiating CRC from IBD or NTC. Different CT scanning phases show different value in distinguishing these disorders. In further studies, we plan to concentrate on standardizing the scanning protocol to validate it on a larger scale before conducting tests in clinical practice.

## Materials and Methods

### Ethics approval

This study was conducted in accordance with the Declaration of Helsinki and approved by the Ethics Committee of the Renji Hospital of Shanghai Jiao Tong University. All participants signed informed consent forms.

### Patient selection

**Figure 4:**
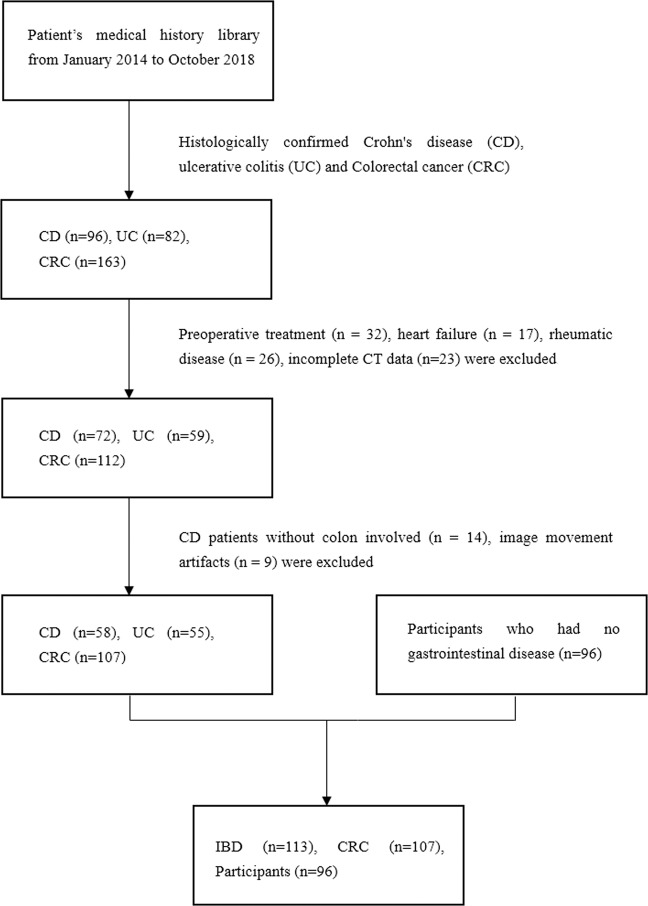
Workflow diagram of patients screening.

This retrospective study was approved by the local institutional review board (IRB), and written informed patient consent was necessary in this retrospective study. To enrol patients with suspected colonic lesions on multiphase contrast-enhanced CT, we first performed a computerized search of the patient medical history library from January 2014 to October 2018. We sequentially enrolled 96, 82 and 163 patients with histologically confirmed CD, UC and CRC, respectively. The histological outcome in these cases was obtained by endoscopic biopsy or surgical resection. Second, we excluded 24 of 96 CD patients, 23 of 82 UC patients and 51 of 163 CRC patients from this study because of preoperative radiotherapy or chemotherapy (n = 32), heart failure (n = 17), rheumatic disease (n = 26), lack of CT (n = 15), only unenhanced CT (n = 3) or only single phase enhanced CT (n = 5). Finally, we excluded an additional 14 patients with CD without colon involvement and 9 patients with movement artefacts in the CT images. The inclusion criteria were as follows: (1) all patients with histologically confirmed CD, UC or CRC; and (2) all patients with complete CT data (PCP, AP and PVP) and clinical information. The exclusion criteria were as follows: (1) patients without enhanced CT scans or with CT image quality that did not meet the requirements; and (2) patients who had received preoperative treatment or suffered from other diseases that may affect the image analysis. Multiphase CT images were separately analysed by two experienced radiologists (J. Z., Q. F.) with 24 and 15 years of experience in diagnostic gastrointestinal imaging. This review resulted in 58 CD patients with colon involvement, 55 patients with UC, and 107 patients with CRC. Thus, there were 113 IBD and 107 CRC patients in total. As a control group, we also included 96 patients with digestive system symptoms who were referred for abdominal multiphase enhanced CT scans but had no abnormal findings. A workflow diagram of this study with respect to patient selection is shown in Fig. 4. The clinical information of these patients is listed in Table 6.

**Table 6:** Clinical and histopathological information about patients with CRC and IBD.

Variable	n (%)
CRC	107
Male	58 (54)
Female	49 (46)
Average age (years)	61.5 ± 9.2
Tumor location	
Ileocecum	17 (15.9)
Ascending colon	14 (13.1)
Transverse colon	19 (17.8)
Descending colon	24 (22.4)
Sigmoid colon	33 (30.8)
T stage	
T1	32 (29.9)
T2	29 (27.1)
T3	27 (25.2)
T4	19 (17.8)
Clinical grade	
Grade I	27 (25.2)
Grade II	58 (54.2)
Grade III	22 (20.6)
Histological type	
Mucinous adenocarcinoma	25 (23.4)
Tubular adenocarcinoma	82 (76.6)
IBD	113
Male	66 (58.4)
Female	47 (41.6)
Average age (years)	36.3 ± 10.6
Duration of IBD (Months)	38.8 ± 59.6
Crohn disease	58 (51.3)
Ulcerative colitis	55 (48.7)
Active IBD	36 (31.9)
Chronic IBD	77 (68.1)

### CT protocol

Abdominal multiphase contrast-enhanced CT was performed on two CT scanners: (1) a 64-channel multidetector CT scanner (Discovery CT750 HD or Lightspeed VCT, GE Healthcare, Milwaukee, USA) and (2) a 128-channel multidetector CT scanner (SOMATOM definition AS + , Siemens Healthcare, Erlangen, Germany). According to the abdominal CT instructions in our department, all patients received a liquid diet and underwent cathartic preparation 24 h before the CT examination. With the patient’s tolerance, 1 to 1.5 L of warm water (30 °C~40 °C) was gently injected through the anus, followed by three consecutive CT scans (with all 3 phases included) with the patient in the supine position. PCP CT was performed covering the entire abdomen from the diaphragmatic dome to the symphysis pubis. Following the PCP CT scan, AP and PVP CT scans were performed sequentially with the same coverage. These two contrast-enhanced CT scans were implemented at 35 s and 60 s, respectively, after 75–150 ml (1.5 ml/kg) of nonionic iodinated contrast agent (370 Iopamidol, Shanghai Bracco Sine Pharmaceutical China) was automatically injected through the antecubital vein at a speed of 3.5 ml/s. The scanning parameters for PCP CT were as follows: 120 kV, 200–350 mA; field of view, 40–50 cm; slice thickness, 1.2 mm or 1.25 mm; interval, 1.2 mm or 1.25 mm; matrix, 512×512; tube rotation time, 0.6 s-0.8 s; pitch, 1–1.375:1; and reconstruction kernel, standard algorithm. After reconstruction, images were displayed with a cross-sectional thickness of 1.0 mm and an in-plane resolution of 0.60 × 0.60 mm. The resulting CT images were reviewed through our institutional picture archiving and communication system server.

### Image selection

To select typical images for TA from each CT scan, the three-phase CT images of each patient’s colon were sequentially viewed from the rectum to the ileocaecal junction following the course of the colon. When CRC lesions or abnormal colonic thickening were localized, three representative axial images of each CT scan were defined. The representative images on the three CT scans (PCP, AP and PVP) were defined at the same cross-section. For CRC, the first axial image was acquired in the middle of the tumour, avoiding any necrosis or blood vessels. The second and third images were taken at the midline between the middle and upper border and between the middle and lower border of the tumour, respectively. For IBD patients (UC and CD) and normal participants, three axial images of the colon were selected in the ascending, transverse and descending colon (including the sigmoid colon) based on the following criteria: (a) the thickness of the thickened colon wall or lesions was more than 5 mm; (b) asymmetrical or localized colonic thickening was preferred; and (c) the thickened colon wall contained lesions in patients with IBD. The CT images were reviewed, and representative images were selected by the previous two gastrointestinal radiologists (J.Z., Q.F.) together, and any disagreements were resolved through consensus. Each of the three selected axial colonic images was anonymized and exported from the picture archiving and communication system.

### TA and classification based on TA

**Figure 5:**
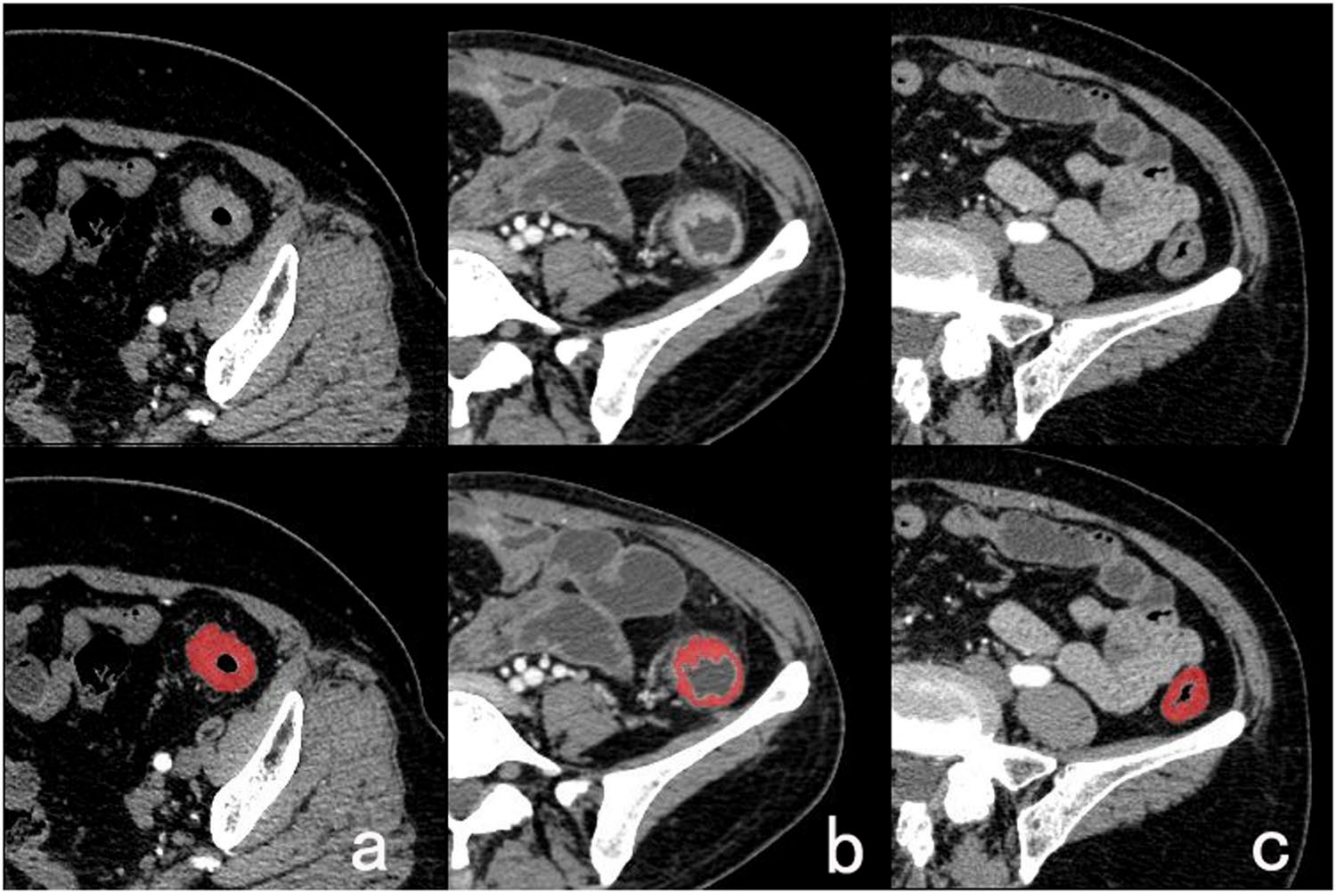
Delineation of Region of Interest (ROI) in arterial phase. (**a**) For colon carcinoma, (**b**) for ulcerative colitis, (**c**) for normal thickened colon wall.

The selected single axial colonic CT images (DICOM format) were transformed into bitmap format images and segmented the lesions by MaZda 4.6 software (http://www.eletel.p.lodz.pl/programy/mazda/). Each image was manually contoured and measured by two independent radiological residents (readers A and B, who had 3 years and 5 years of experience in diagnosis, respectively) to define the outer margin of the thickened colon wall or lesion and was saved as a ROI for further TA (Fig. 5 CRC in AP (a), IBD in AP (b) and NTC in AP (c)). The two radiological residents were blinded to the pathological results of these patients. The outline was drawn slightly within the thickened colon wall (for IBD patients and normal participants) or the tumour borders to eliminate volume effects of the adjacent pericolonic fat or gas. Taking into account that the boundaries of the colon can be difficult to identify from a non-enhanced CT scan in some patients or participants, the corresponding enhanced images could be used to define the outline. Each reader recorded the pixels contained in each contoured ROI and the maximum thickness of the thickened colon wall or tumour (reading A1 and B). Reader A contoured the ROI again 4 weeks later to investigate the internal consistency of the observer (reading A2). The obtained contours from readings A1, A2, and B were analysed for texture by an independent reviewer.

Before TA, the grey scale of every contoured ROI was normalized with a dynamic limitation of µ ± 3δ (µ, mean; δ, standard deviation) to minimize the effects of contrast and brightness variation, which might otherwise blur the real texture^32^. After normalization, texture features were calculated using image processing techniques, including the grey histogram, the run-length and co-occurrence matrix, the absolute gradient, the autoregressive model, and the wavelet transform (see Supplementary Table C). To determine which texture features were most useful for distinguishing CRC, inflammatory lesions of IBD and NTC from the control, the previously calculated texture features were further extracted by the Fisher coefficient, POE + ACC and MI coefficient^31^. The program B11 (http://www.eletel.p.lodz.pl/programy/cost/projekt_cost.html), which studies data to decrease the vector dimension and increase the discriminatory value, was used for the statistical evaluation of features. We used three different approaches in program B11: (i) PCA; (ii) LDA; (iii) NDA. The features extracted from PCA, LDA were further classified by k-NN classifier and the features extracted from NDA was classified by the ANN classifier, respectively. Data vector misclassification by k-NN and ANN for the differentiation of CRC, IBD lesions and NTC was studied separately for PCP, AP and PVP images.

To test intra- (reader A1 and A2) and inter-observer (reader A1 and B) consistency in the selection of texture features, the texture features selected using the following methods for each reader and the reproducibility of these features were analysed: grey-level histogram mean and variance, angular second moment, entropy, total entropy, difference in variance, difference in entropy from the co-occurrence matrix, and difference in run-length and grey scale from the run-length matrix. The definitions of the texture features are summarized in Supplementary Table D.

### Visual classification

All CT images of each patient were reviewed by two attending gastrointestinal radiologists (readers C and D) with 12 and 10 years of experience and two young residents (readers E and F) with 3 and 4 years of experience, respectively. The readers were blinded to the patient information, including the pathological and TA results. In visual analysis, the readers set the optimal window and level according to visual feedback to ensure sufficient lesion visibility. One scanning phase was reviewed each time. Two weeks later, the next scanning phase was reviewed to avoid memory effects. Readers independently made the diagnosis of CRC, IBD or NTC mainly based on the pattern of colon wall thickening and lesion contrast-enhancement characteristics. The MCR of visualization for each gastrointestinal radiologist was calculated according to the following equation:

$${\rm{MCR}}\,( \% )=\left(1-\frac{Number\,of\,cases\,with\,correct\,diagnosis}{Number\,of\,all\,cases}\right)\times 100 \% $$

### Statistical analysis

The number of pixels in the ROI and the thickness of the colon wall are expressed as the mean ± SD. Our analysis was limited to patient-level means for each feature and for each set of contours (A1, A2, and B). The measurement differences among readings (A1, A2, and B) in the same images were analysed by analysis of variance (ANOVA). Intra-observer (A1, A2) and inter-observer (A1, B) agreement between the ROI pixels and thickness measurement sessions were assessed with the ICC. An ICC of 0–20, 20–40, 40–60, 60–80, and 80–100 indicated poor, fair, moderate, substantial agreement and very good agreement, respectively. The repeatability of textural features within (A1 vs A2) and between (A1 vs B) readers was assessed with the concordance coefficient (Rc) and were displayed graphically using the Bland-Altman method. A Rc of <0.90, 0.90–95, 0.95–0.99 and >0.99 indicated poor, moderate, substantial and almost perfect agreement, respectively. Mann-Whitney U tests were performed to compare the MCR for the differentiation of CRC, IBD and NTC in each CT scanning phase between the CTTA and the visual analysis. Statistical analysis was performed using SPSS software (version 22.0), and p values less than 0.05 were considered to indicate significant differences. The classification capability of the calculated texture features was evaluated by ROC curve analysis using MedCalc software (vision 19.1.7, MedCalc Software, Ltd., Ostend, Belgium).

### Ethical approval

All procedures performed in studies involving human participants were in accordance with the ethical standards of the institutional and/or national research committee and with the 1964 Helsinki declaration and its later amendments or comparable ethical standards.

### Informed consent

Informed consent was obtained from all individual participants included in the study.

## Supplementary information

Supplementary Tables
