# Supplementary material for: The feasibility of differentiating colorectal cancer from normal and inflammatory thickening colon wall using CT texture analysis
Source: Sci Rep. 2020 Apr 14;10:6346. doi: 10.1038/s41598-020-62973-1 (PMC7156692; doi:10.1038/s41598-020-62973-1)
Supplement: Supplementary file 1 — Supplementary Tables [file 41598_2020_62973_MOESM1_ESM.pdf]

The feasibility of differentiating colorectal cancer from normal and inflammatory thickening colon wall using CT texture analysis

Authors

Xiao Wang, Mingyuan Yuan, Honglan Mi, Shiteng Suo, Khalid Eteer, Suqin Li, Qing Lu, Jianrong Xu, Jiani Hu

Supplementary Table A. Intra-and inter-observer concordance and repeatability of extracted texture features.

Texture features	A1 vs A2		A1 vs B	
	R_C	95% confidence interval	R_C	95% confidence interval
Mean	0.99	0.98, 0.99	0.91	0.90, 0.93
Variance	0.99	0.98, 0.99	0.94	0.94, 0.96
Angular second moment	0.95	0.95, 0.96	0.93	0.93, 0.94
Sum entropy	0.92	0.90, 0.93	0.93	0.91, 0.94
entropy	0.95	0.92, 0.96	0.92	0.90, 0,93
Difference variance	0.99	0.98, 0.99	0.91	0.90, 0.93
Difference entropy	0.95	0.94, 0.95	0.91	0.89, 0.92
Run-length non-uniformity	0.98	0.97, 0.98	0.92	0.89, 0.93
Gray-level non-uniformity	0.99	0.98, 0.99	0.93	0.91, 0.94

Note: A1, first read of reader A; A2, second read of reader A; B, reader B; Rc, concordance coefficients; Rc value of < 0.90, 0.90–95, 0.95-0.99 and > 0.99 indicated poor, moderate, substantial and almost perfect agreement.

Supplementary Table B. ROC curves analysis of extracted texture features.

	Texture features	AUC	95% Confidence Interval
CRC vs. IBD	Mean	0.90±0.03	0.84, 0.94
	Variance	0.89±0.03	0.83, 0.94
	Angular second moment	0.88±0.03	0.81, 0.93
	Sum entropy	0.87±0.02	0.80, 0.92
	Entropy	0.87±0.03	0.80, 0.92
	Difference variance	0.88±0.02	0.82, 0.93
	Difference entropy	0.88±0.04	0.82, 0.93
	Run-length nonuniformity	0.87±0.03	0.80, 0.92
	Gray-length nonuniformity	0.85±0.03	0.78, 0.90
	All 9 features	0.94±0.02	0.89, 0.97
IBD vs. NTC	Mean	0.89±0.03	0.83, 0.93
	Variance	0.89±0.03	0.83, 0.94
	Angular second moment	0.88±0.02	0.81, 0.92
	Sum entropy	0.87±0.03	0.80, 0.91
	Entropy	0.87±0.03	0.80, 0.91
	Difference variance	0.88±0.02	0.81, 0.92
	Difference entropy	0.88±0.03	0.82, 0.93
	Run-length nonuniformity	0.87±0.02	0.80, 0.91
	Gray-length nonuniformity	0.86±0.03	0.80, 0.91
	All 9 features	0.97±0.01	0.92, 0.98
CRC vs. NTC	Mean	0.93±0.03	0.87, 0.97
	Variance	0.95±0.03	0.88, 0.98
	Angular second moment	0.94±0.03	0.87, 0.97
	Sum entropy	0.93±0.03	0.86, 0.96
	Entropy	0.91±0.02	0.84, 0.95
	Difference variance	0.92±0.03	0.85, 0.96
	Difference entropy	0.94±0.03	0.87, 0.97
	Run-length nonuniformity	0.92±0.03	0.85, 0.96
	Gray-length nonuniformity	0.91±0.03	0.84, 0.95
	All 9 features	0.98±0.01	0.96, 0.99
CRC vs. IBD vs. NTC	Mean	0.88±0.03	0.81, 0.93
	Variance	0.86±0.03	0.78, 0.91
	Angular second moment	0.88±0.03	0.81, 0.93
	Sum entropy	0.88±0.03	0.80, 0.92
	Entropy	0.88±0.02	0.81, 0.92
	Difference variance	0.88±0.03	0.80, 0.92
	Difference entropy	0.88±0.03	0.80, 0.92
	Run-length nonuniformity	0.86±0.03	0.78, 0.91
	Gray-length nonuniformity	0.86±0.03	0.78, 0.91
	All 9 features	0.92±0.02	0.86, 0.96

Supplementary Table C. List of texture features.

The gray-level histogram	Mean, Variance, Skewness, Kurtosis, Percentiles (1, 10, 50, 90, 99%)
The co-occurrence matrix (COM)	Angular second moment, Contrast, Correlation, Sum of squares, Sum average, Sum variance, Sum entropy, Entropy, Inverse difference moment, Difference variance, Difference entropy (20 pairs of pixels in 4 directions)
The run-length matrix (RLM)	Short run emphasis inverse moment, Long run emphasis moment, Gray-level non-uniformity, Run length non-uniformity, Fraction of image in runs (4 directions: horizontal, vertical, at 45° and at 135°)
The absolute gradient (GrM)	Gradient mean, variance, skewness, kurtosis, Percentage of pixels with non-zeros gradient
The autoregressive model (ARM)	Theta1-4, Sigma
The wavelet transform	Energy of wavelet transform coefficients in subbands LL, LH, HL, HH

Supplementary Table D. Formulas used to calculate the various texture features.

Texture features	Formula
Mean	$\sum_{i=1}^{N_g} ip(i)$
Variance	$\sum_{i=1}^{N_g} (i-\mu)^2 p(i)$
Angular second moment	$\sum_{i=1}^{N_g}\sum_{j=1}^{N_g} p(i,j)^2$
Sum entropy	$-\sum_{i=1}^{2N_g} p_{x+y}(i)\log(p_{x+y}(i))$
Entropy	$-\sum_{i=1}^{N_g}\sum_{j=1}^{N_g} \frac{p(i,j)}{R}\log(\frac{p(i,j)}{R})$
Difference variance	$\sum_{i=0}^{N_{g-1}} (i-\mu_{x-y})^2 p_{x-y}(i)$
Difference entropy	$-\sum_{i=1}^{N_g} p_{x-y}(i)\log(p_{x-y}(i))$
Run-length nonuniformity	$(\sum_{j=1}^{N_r}(\sum_{i=1}^{N_g} p(i,j))^2)/C$
Gray-length nonuniformity	$(\sum_{i=1}^{N_g}(\sum_{j=1}^{N_r} p(i,j))^2)/C$

Note: Where *P (i, j)* indicates the joint probability of two pixels having particular co-occurring values *i,j* = 1, 2, …, *R* indicates the total number of neighboring pixel pairs, and μ indicate means and standard deviations of the row and column sums of the co-occurrence matrix. *Ng* and *Nγ* are the number of pixels, and *Ng* is the number of distinct gray levels.
